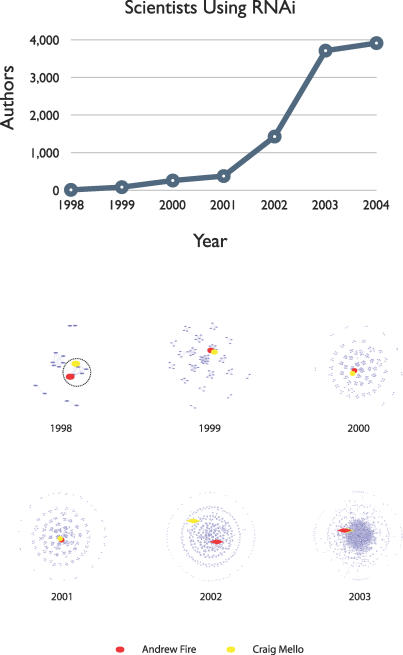# RNAi Development

**DOI:** 10.1371/journal.pcbi.0030080

**Published:** 2007-04-27

**Authors:** Mark Gerstein, Shawn M Douglas

The Nobel Prize in Physiology or Medicine in 2006 was awarded to Craig Mello and Andrew Fire for the development of essentially a new field, RNA Interference or RNAi. Because this field sprung from a singular discovery made very recently, we can track its growth in precise detail in the biological and literature databases. In particular, in the figure we show the results for searching PubMed for the term “RNA Interference” in each of the years from 1998 to 2003. (This was done with a tool called PubNet that allows one to visualize the networks generated by arbitrary queries against the National Center for Biotechnology Institute's PubMed database [1].) The top subgraph simply shows that the term first appeared in 1998, when RNAi was definitively characterized in Caenorhabditis elegans; then there was a rapid increase in the number of authors using the term, particularly around 2001. The bottom subgraph shows authors (represented by nodes) who are linked together when they published together in a given year. It dramatically illustrates that in 1998 there were a small number of distinct author clusters; one of these, highlighted by a dotted line in the figure, corresponded to the classic effort of Fire and Mello in *Nature* [2], describing the phenomena of degradation of double-stranded RNA. In subsequent years, one can see that Fire and Mello continued to publish together as a collaborative unit, but many additional groups of investigators appeared, with the number of new groups increasing very rapidly from 1999 to 2001. However, in 2002, Fire and Mello separated and became part of two disconnected publication clusters. Finally, in 2003, one sees a new phenomenon: there were so many authors in the field that they all merged into a huge mega-cluster. Fire and Mello were again connected in the framework of this cluster. That is, there were so many authors in the RNAi field that everyone was linked to everyone else through at least one co-publication event. In a sense, one is witnessing a “social phase transition”: in just five years, a singular discovery spread through the scientific community, progressed through a “tipping point,” and became commonplace.

**Figure:**